# A novel class of heat-responsive small RNAs derived from the chloroplast genome of Chinese cabbage (*Brassica rapa*)

**DOI:** 10.1186/1471-2164-12-289

**Published:** 2011-06-03

**Authors:** Lu Wang, Xiang Yu, Han Wang, Yi-Zhen Lu, Marjo de Ruiter, Marcel Prins, Yu-Ke He

**Affiliations:** 1 National Key Laboratory of Plant Molecular Genetics, Institute of Plant Physiology and Ecology, Shanghai Institutes for Biological Sciences, Chinese Academy of Sciences, 300 Fenglin Road, Shanghai 200032, China; 2 Keygene, N.V., Agro Business Park 90, 6708 PW Wageningen, The Netherlands; 3 James D. Watson Institute of Genome Sciences, Zhejiang University, 268 Kaixuan Road, Hangzhou 310012, China; 4 T-Life Research Center Fudan University, Shanghai 200433, China

## Abstract

**Background:**

Non-coding small RNAs play critical roles in various cellular processes in a wide spectrum of eukaryotic organisms. Their responses to abiotic stress have become a popular topic of economic and scientific importance in biological research. Several studies in recent years have reported a small number of non-coding small RNAs that map to chloroplast genomes. However, it remains uncertain whether small RNAs are generated from chloroplast genome and how they respond to environmental stress, such as high temperature. Chinese cabbage is an important vegetable crop, and heat stress usually causes great losses in yields and quality. Under heat stress, the leaves become etiolated due to the disruption and disassembly of chloroplasts. In an attempt to determine the heat-responsive small RNAs in chloroplast genome of Chinese cabbage, we carried out deep sequencing, using heat-treated samples, and analysed the proportion of small RNAs that were matched to chloroplast genome.

**Results:**

Deep sequencing provided evidence that a novel subset of small RNAs were derived from the chloroplast genome of Chinese cabbage. The chloroplast small RNAs (csRNAs) include those derived from mRNA, rRNA, tRNA and intergenic RNA. The rRNA-derived csRNAs were preferentially located at the 3'-ends of the rRNAs, while the tRNA-derived csRNAs were mainly located at 5'-termini of the tRNAs. After heat treatment, the abundance of csRNAs decreased in seedlings, except those of 24 nt in length. The novel heat-responsive csRNAs and their locations in the chloroplast were verified by Northern blotting. The regulation of some csRNAs to the putative target genes were identified by real-time PCR. Our results reveal that high temperature suppresses the production of some csRNAs, which have potential roles in transcriptional or post-transcriptional regulation.

**Conclusions:**

In addition to nucleus, the chloroplast is another important organelle that generates a number of small RNAs. Many members of csRNA families are highly sensitive to heat stress. Some csRNAs respond to heat stress by silencing target genes. We suggest that proper temperature is important for production of chloroplast small RNAs, which are associated with plant resistance to abiotic stress.

## Background

In eukaryotes, small non-protein-coding RNAs have emerged as key guidance molecules that fulfill important, vital functions in many cellular processes, such as transcription, translation, splicing, DNA replication and RNA processing [1]. It is thought that small RNAs are produced by Dicer-like proteins (DCLs) from their precursors, which can be stem-loop RNA transcripts or long double-stranded RNAs (dsRNAs). The small RNAs directly interact with proteins from the Piwi/Argonaute (AGO) family to form the core of the RNA induced silencing complex (RISC) [2,3]. However, data collected from animals suggest a correlation between small RNA diversity and morphological complexity [4,5]. Recent progress in the understanding of the non-canonical mechanisms of small RNA biogenesis has been achieved in mammalian systems [6]. Massive amounts of data produced by next generation sequencing technologies also revealed subclasses of small non-coding RNA species that were derived by alternative biogenesis pathways and only partially met classical definitions, such as small RNAs derived from genomic loci containing repeat sequences [7], snoRNA [8] and tRNA [9]. Although most of these studies focused on nuclear or cytosolic members, small ncRNAs from subcellular genomes, such as chloroplasts and mitochondria, have gradually risen into view in animals, plants and fungi [10-12].

Chloroplasts and mitochondria, widely accepted as endosymbiontic eubacteria in cells, retain separate circular genomes, and their own gene expression machinery [13]. One hallmark of organellar genomes is the predominance of post-transcriptional control, including RNA processing and turnover, which are exerted both at the gene-specific and global levels [14-16]. In chloroplasts, a small number of non-coding RNAs have been reported previously. For example, a 218-nt-long plastid-encoded RNA is thought to be relevant to the maturation of 16S ribosomal RNA [17] that was initially discovered in tobacco and then found to be conserved in several other angiosperms, and 12 ncRNAs have been identified in tobacco chloroplast with unknown functions, some of which were predicted to form possible stem-loop structures [10]. In chloroplasts, nearly all polycistronic transcripts are processed by endonucleases or/and exonucleases [18], which may relieve secondary structures for ribosome assembly [14]. Also, 3' end maturation in chloroplasts follows a prokaryotic pathway with rho-independent termination, resulting in mature termini flanking stem-loop structures [19]. Nevertheless, RNA processing is still poorly understood in chloroplast, compared to that of well-studied *Escherichia coli*. The enzymatic machinery is only beginning to be explored, and its molecular nature remains unclear.

Chinese cabbage (*Brassica rapa *ssp. *chinensis*) is one of the most widely grown leafy vegetables, and its plant is composed of numerous green leaves, which contain abundant chloroplasts. This botanical characteristic, along with its close genetic relationship to *Arabidopsis*, make it suitable material for the study of chloroplast development. The adaptable growth temperature for Chinese cabbage ranges from 18°C to 22°C, and its production is seriously threatened by heat stress in many regions. Heat stress causes a visible growth inhibition of shoot and root [20]. One of the phenotypes is leaf etiolation and bleaching. In the etiolated leaf segments, a clear-cut inhibition of photosynthetic activity, chlorophyll accumulation and chloroplast development can be observed [21], thus indicating a strong response of chloroplasts to heat stress. In recent years, a great deal of attention has been paid to the elucidation of the mechanisms of heat-sensitivity for breeding heat-resistant cultivars of Chinese cabbage and other important crops. Considering the importance of small RNAs in abiotic stress resistance [22-24] and their advantage in terms of regulating the plurality of target genes rather than one single gene and existence of small RNAs in the chloroplast [10], we were inspired to search for small RNAs in chloroplast associated with heat resistance of Chinese cabbage.

In this study, we report a novel class of chloroplast small RNAs (csRNAs) in Chinese cabbage and *Arabidopsis*. Significant numbers of csRNAs are responsive to high temperature. These novel csRNAs may play potential roles in the heat response, probably by regulating chloroplast RNA processing and target gene expression.

## Results

### Leaf etiolation under heat stress

**Figure 1:**
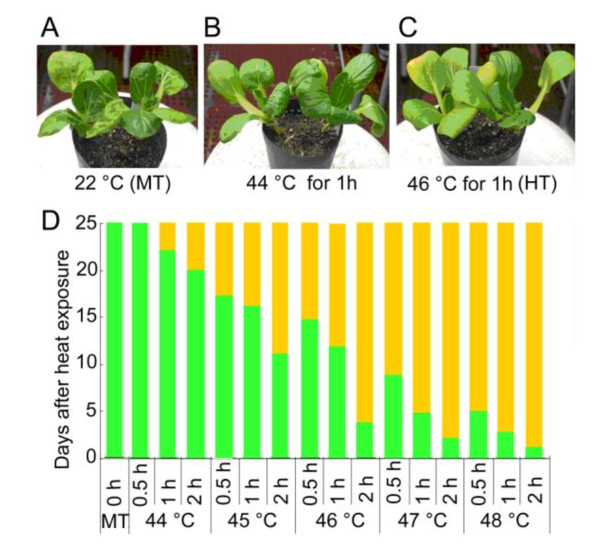
**Seedlings of Chinese cabbage are etiolated under heat stress**. (**A**) A seedling growing 20 days after treatment at 22°C for 1 h (MT). (**B**) A seedling growing 20 days after treatment at 44°C for 1 h. (**C**) A seedling growing 20 days after treatment at 46°C for 1 h (HT). (**D**) Green color indicates the green leaves after HT treatment while yellow color shows the etiolated leaves after HT treatment.

To define the effects of high temperature on leaf etiolation, we incubated the seedlings of non-heading Chinese cabbage at 44, 45, 46, 47 and 48°C for durations of 0.5, 1 and 2 hours. As indicated in Figure 1, the seedlings exposed to 44°C for 0.5 h grew as normally as those exposed to 22°C, while the seedlings exposed to 44°C for 1 hour grew more slowly than those exposed to 22°C and were etiolated on the 22^nd ^day after the heat treatment. This indicated that the heat treatment of 44°C for 1 hour caused morphological and physiological change in the seedlings. As the temperature rose and the heat treatment prolonged, the inhibitory effects on plant growth became more severe and leaf etiolation appeared earlier and occurred to a more significant degree (Figure 1D). We noticed that the seedlings exposed to 46°C for 1 h were etiolated on the 12^th ^day after the heat treatment (Figure 1C), while those exposed to 47°C for 1 h etiolated on the 5^th ^day after heat treatment, revealing that 47°C for 1 h is a critical temperature and time point for this genotype. Considering the differences in the early gene expression events and physiological reactions observed in the plants, we chose the heat treatment of 46°C (HT) and the moderate temperature (MT) for the small RNA deep sequencing experiments. We expected that the temperature of this heat treatment was high enough to affect the biogenesis of small RNAs but did not cause significant morphological and physiological changes to the seedlings. The heat-treatment method is simple, easy to manage, accurate and only required a short length of time. Using this method, we have selected more than 20 lines of Chinese cabbage with heat resistance.

### Small RNAs generated from the chloroplast genomes of Chinese cabbage

For a global view of small RNAs expressed in Chinese cabbage, we chose to collect material from the above ground portions of HT and MT seedlings and sequenced RNA fractions of 9 to 36 nucleotides (nt), using Illumina Genome Analyzer and mirVana™ miRNA Isolation Kit (Ambion, Inc). Two replicates of HT treatment generated 14.6 million and 12.8 million small RNA reads, while those of the MT treatment produced 14.7 and 11.3 million small RNA reads, indicating high quality achieved. For a convenient comparison, the data from HT with 14.6 million and that from MT with 14.7 million reads were used for the analysis.

**Figure 2:**
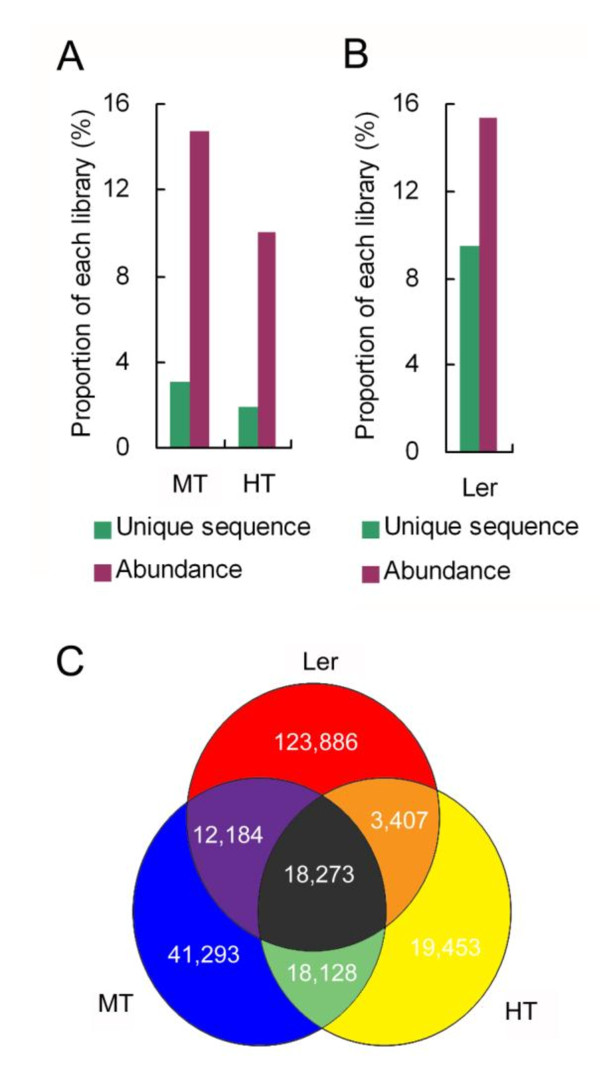
**Proportion of csRNAs in each sequence dataset**. (**A**) Percentages of Chinese cabbage csRNAs. (**B**) Percentages of *Arabidopsis *csRNAs. (**C**) Venn diagram of Chinese cabbage csRNAs and *Arabidopsis *csRNA datasets. The numbers represent the number of unique csRNAs in each plot.

To select chloroplast small RNAs from the whole population of reads, the trimmed sequences were compared to a complete chloroplast genome and a nuclear genome of Chinese cabbage by BLAST after the removal of the adapters. The small RNAs that perfectly-matched the chloroplast genome were retained. In total, we obtained 2,162,001 and 1,278,752 csRNAs from MT and HT, respectively, which represents 116,210 and 78,299 unique small RNA sequences, and account for 15% and 10% of the total small RNA population, respectively (Figure 2A and Table 1). Since the chloroplast genome sequence of Chinese cabbage had not been annotated, we matched all small RNA sequences with the *Arabidopsis *chloroplast genome for further genomic origin analyse. The two species share 98% identity in the DNA sequences of their chloroplast genomes. In this case, more than 95% of the small RNAs from Chinese cabbage are matched perfectly with the *Arabidopsis *chloroplast genome (Table 1). The small RNAs that are perfectly matched with both genomes of two species were designated as chloroplast-related small RNAs (csRNAs).

**Table 1:** Chinese cabbage Small RNA reads originated from chloroplast genome

*Dataset*	*Origin*	*Unique sequence (Reads)*	*Abundance (Reads)*
	Total	4,439,626	14,622,224
	
HT	Mapped to the Chinese cabbage chloroplast genome	78,299	1,278,752
	
	Mapped to both Chinese cabbage and *Arabidopsis *chloroplast genomes ^a^	59,261	1,225,538

	Total	3,816,252	14,690,798
	
MT	Mapped to the Chinese cabbage chloroplast genome	116,210	2,162,001
	
	Mapped to both Chinese cabbage and *Arabidopsis *chloroplast genomes	89,878	2,095,078

^a^: Chinese cabbage csRNA dataset

Since some fractions of chloroplast DNA sequences match the nuclear or mitochondrial genome, the csRNAs should include those from the nucleus and mitochondria. To estimate the proportion of chloroplast-specific small RNAs that originated from chloroplast genome rather than nuclear and mitochondrial genomes, we established the chloroplast-related small RNA dataset of *Arabidopsis thaliana *(Landsberg erecta ecotype) paralleled to that of Chinese cabbage by small RNA deep-sequencing, and then calculated the abundance of the csRNA sequences in the *Arabidopsis *chloroplast genome. In total, 787,272 csRNA sequences from *Arabidopsis *perfectly matched its chloroplast genome (Additional File 1). By removing those sequences that matched the nuclear and/or mitochondrial genomes, we obtained the chloroplast-specific small RNAs of *Arabidopsis*, which account for 86% of the unique csRNAs. The size distribution and the origin composition of csRNAs were consistent with those of chloroplast-specific small RNAs (Additional File 2). A high proportion of chloroplast-specific small RNAs and the similar compositions revealed that the csRNA dataset is representative of its chloroplast-specific small RNA dataset. Considering the close relation between two species, the property of csRNAs in the Chinese cabbage dataset should be representative of the entire population of chloroplast-derived small RNAs.

### The distribution of csRNAs over the genome

**Figure 3:**
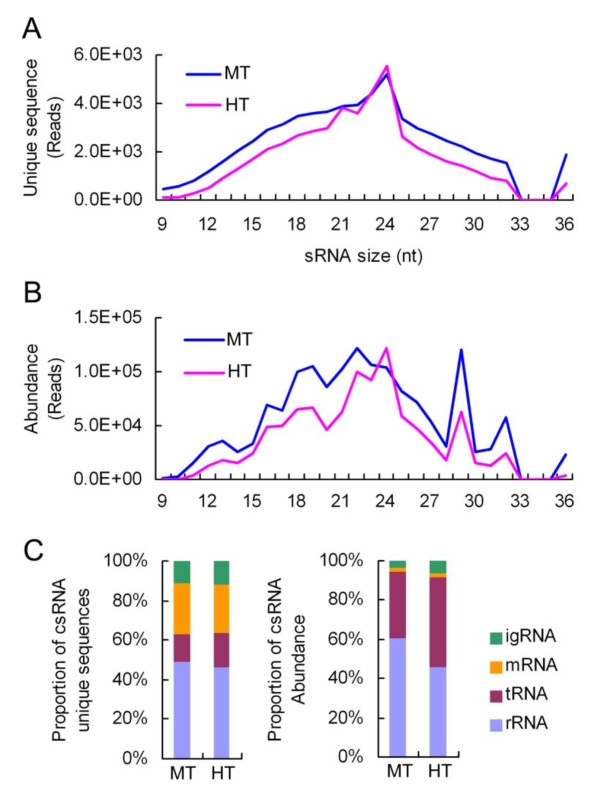
**Size distribution of Chinese cabbage csRNAs and origin of RNA**. (**A**) Size distribution of unique csRNAs of Chinese cabbage. (**B**) Size distribution of total csRNAs of Chinese cabbage. (**C**) RNA origin of csRNA population of Chinese cabbage.

csRNAs of Chinese cabbage exhibited wide variations in length, from 9 to 36 nt (Figure 3A and 3B). Among the small RNAs mapped to the chloroplast genome, the most numerous unique csRNAs were 24 nt in length (Figure 3A), but 22 nt small RNAs were the most abundant in MT seedlings (Figure 3B). csRNAs were classified into four types: mRNA, rRNA, tRNA, and intergenic RNAs (igRNA), according to the types of RNA from which csRNAs are derived. Among them, the csRNAs derived from rRNA are the most abundant, and those from mRNA are the least prevalent (Figure 3C and Additional File 3). Further analysis showed that a large proportion of rRNA-derived csRNAs were 18 to 25 nt in length with three peaks of 18-19, 21-22 and 25 nt, while the csRNAs derived from tRNA showed a maximum of 29 nt with 2 smaller peaks at 23-24 and 32 nt (Additional File 4). In addition, mRNA-derived csRNAs are generally 21-24 nt in length (Additional File 4E and 4F), similar to that reported by the early work on nuclear-expressed genes. The csRNAs originating from igRNAs were on average 22 nt in length, which roughly match the numbers of unique csRNAs (Additional File 4H and 4G). We noticed that some csRNA families were very large as they shared the same core sequence, typically 9-15 nt in length, with 5' or 3' extensions of variable lengths (Additional File 5). This feature did not fit with our canonical knowledge of small RNA biogenesis, as the precursor that would be loaded into the Dicer-like complex seems variable in size.

### csRNAs from 3' ends of rRNA and 5' ends of tRNA

**Figure 4:**
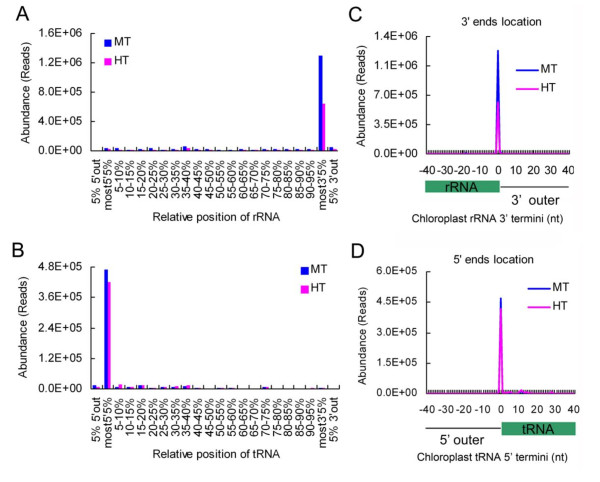
**position-of-origin analysis of Chinese cabbage csRNA**. To normalize the varying rRNAs or tRNAs length, RNA sequences were divided into 5% blocks from the 5' to the 3' ends. The flanking sequence outer of each ends were calculated proportionally. The positions of csRNA 3' ends and 5' ends were plotted into rRNA (**A**) and tRNA (**B**) blocks, respectively. (**C**) The peak of 3' ends of rRNA-derived csRNAs at the 3' termini of rRNA. (**D**) The peak of 5' ends of tRNA-derived csRNAs at the 5' termini of tRNA.

The chloroplast genome contains two inverse repeats, from where all fragments of rRNA are transcribed [25]. Each repeat consists of four types of rRNA: 23S, 16S, 5S and 4.5S. To examine the distribution of csRNAs along rRNA sequences, we divided the transcribed regions of rRNA into 5% blocks from the 5' to 3' ends, and plotted the positions of csRNA ends into them. Interestingly, an enrichment of the rRNA-derived-csRNAs were fallen into the last 5% block of chloroplast rRNA (Figure 4A), even showing a remarkable peak at the 3' ends of rRNA (Figure 4C). csRNAs from 4.5S rRNA sequences account for a substantial portion of csRNAs originating from rRNA (~70%). An enrichment of the csRNAs from 4.5S and 5S rRNA were fallen into the last 5% block of 4.5S rRNA and 5S rRNA, while those from 16S and 23S rRNA were not (Additional File 6A-D). Moreover, there was a remarkable abundance peak for the last nucleotide of the mature 4.5S rRNA and one outer nucleotide of the mature 5S rRNA 3' terminus, respectively (Additional File 6EE-H), indicating that the biogenesis of csRNAs in the 4.5S and 5S rRNA molecules are 3' end-preferential.

There are 20 types of tRNAs in the chloroplast, representing the entire amino acid (anti) codons [25]. Similarly, we divided the nucleotide sequences of the tRNAs into 5% blocks from the 5' to 3' termini, and plotted the positions of csRNA ends. Interestingly, the csRNAs derived from most of the chloroplast tRNA sequences (except tRNA-Ser, tRNA-Thr and tRNA-Trp) exhibited an abundant peak in the first 5% block, which accounted for more than 95% of the tRNA-derived csRNAs (Figure 4B). Moreover, the first nucleotide of these csRNAs were predominantly located at the first nucleotide of the mature chloroplast tRNA sequences (Figure 4D), revealing that the biogenesis of csRNA in tRNA molecules is specific to the 5' end.

These results are consistent with the previous reports, in which the majority of small RNAs originated from the rDNA locus [26] and the 3' ends of rRNA [27], and the 5' and 3' end-specific small RNAs derived from tRNAs [9] as well as some new classes of small heterogeneous transcripts mapped to the 5' ends of known protein-coding genes have been reported in *Arabidopsis *[28], *Drosophila*, chicken and humans [8].

### Heat response of csRNAs

**Figure 5:**
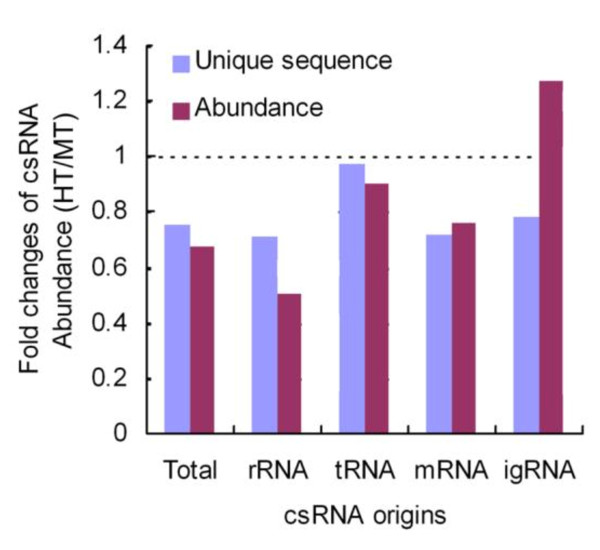
**Fold change of unique sequences and the abundance of Chinese cabbage csRNAs in the HT seedlings**.

On a subcellular level, heat stress has serious consequences for chloroplast activity and structure [29], for example, the loss of chloroplast Cu/Zn superoxide dismutase and increased damage to photosystem II [30]. In the seedlings exposed to 46°C for 1 hour (HT), csRNA abundance declined significantly compared to that of MT seedlings, accounting for approximately 25% of unique sequences and 36% of the csRNA abundance reads in the MT dataset (Figure 5). Additional File 7 lists the down-regulated csRNAs in HT seedlings (less than one-fifth), Additional File 8 shows up-regulated ones (more than 5-fold), and Additional File 9 shows the miRNA microarray analysis of the csRNAs that were remarkably changed after heat treatment.

Among heat-responsive csRNAs, the rRNA-derived csRNAs (rRNA csRNAs) were the most affected. Under heat stress, the abundance of rRNA csRNAs was reduced to approximately 49%, while the number of the unique csRNAs was reduced to approximately 29% (Figure 5 and Additional File 3). Specifically, the abundance of the csRNAs derived from each rRNA were drastically reduced in HT seedlings, and the rRNA-csRNAs of various length were affected (Additional File 10), showing the broad sensitivity to heat stress.

**Figure 6:**
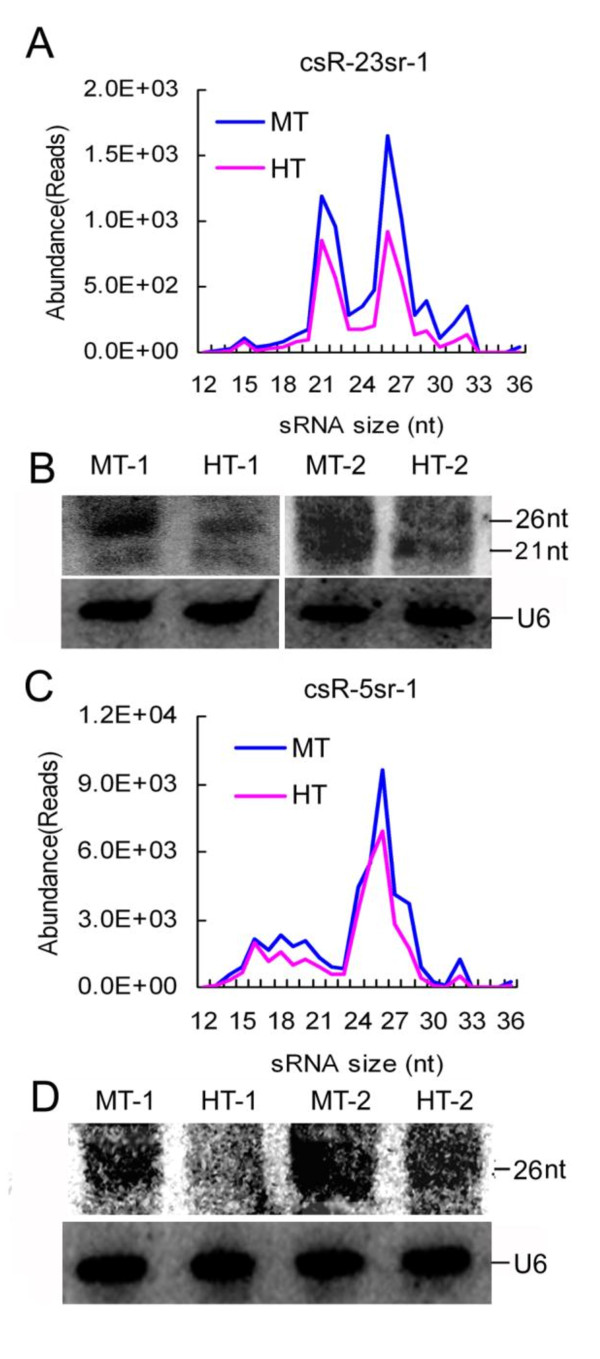
**Identification of csR-23sr-1 and csR-5sr-1**. (**A**) and (**C**) Size distribution analysis of the csR-23sr-1 family and the csR-5sr-1 family in sequencedatasets with core sequences (underlined in Additional File 9). (**B**) and (**D**) Northern blotting analysis of csR-23sr-1 and csR-5sr-1 in RNA samples from heat-treated and control seedlings of Chinese cabbage. U6 spliceosomal RNA was used as an RNA loading control.

We chose two families of the most abundant heat-reduced rRNA csRNAs, csR-5sr-1 and csR-23sr-1, for further analyses. csR-23sr-1 and csR-5sr-1 are derived from 23S and 5S rRNAs, respectively, (sequences in Additional File 9). In Chinese cabbage, csR-23sr-1 are predominantly 26 and 21 nt, while csR-5sr-1 are typically 26 nt in length, and they were reduced after heat treatment (Figure 6A and 6C). To test how heat stress affects accumulation of csR-5sr-1 and csR-23sr-1 csRNAs, we performed Northern hybridization of small RNAs. In the HT seedlings, 26 nt csR-23sr-1 and 26 nt csR-5sr-1 csRNAs accumulated much less than in the MT seedlings (Figure 6B and 6D). These data suggest that accumulation of rRNA-derived csRNAs is affected by heat stress.

**Figure 7:**
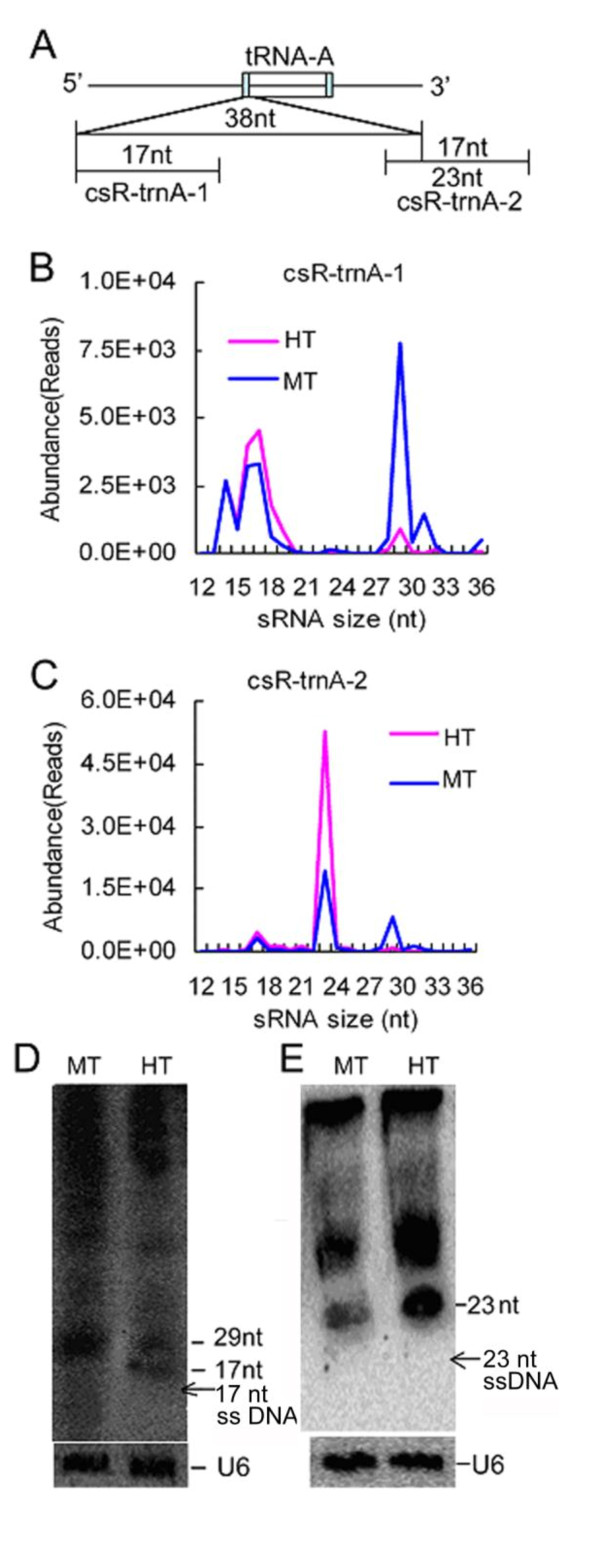
**Identification of csR-trnA-1 and csR-trnA-2**. (**A**) An diagram showing the location of csR-trnA-1 and csR-trnA-2 in chloroplast tRNA-Ala. Two exons of tRNA-A are indicated by boxes. (**B**) and (**C**) Size distribution of the csR-trnA-1 family and the csR-trnA-2 family in sequence datasets with core sequences (underlined in Additional File 10). (**D**) and (**E**) Northern blotting of csR-trnA-1 and csR-trnA-2 in the HT seedlings of Chinese cabbage. U6 spliceosomal RNA was used as an RNA loading control.

As a whole, the tRNA-derived csRNAs only slightly declined in the HT seedlings compared to those in the MT seedlings (Figure 5). However, the length of these csRNAs was related to heat response (Additional File 4C and 4D). In the HT seedlings, the abundance of the longer csRNAs (29-32nt) decreased, but that of the shorter ones (16-25nt) increased compared to those in the MT seedlings (Table 2). For each tRNA, abundance of the short csRNAs increased while that of the longer csRNAs declined except for tRNA-Gly. csR-trnA-1 and csR-trnA-2 are two most predominant tRNA-derived csRNA families, and originated from chloroplast tRNA-Ala (Figure 7A). The former showed two peaks (17 and 29 nt) while the latter displayed only one peak (23 nt) (Figure 7B and 7C) (sequences in Additional File 9). Northern blotting showed that abundance of 29 nt csR-trnA-1 csRNAs under heat stress declined while those of 17 nt csRNAs increased (Figure 7D), and abundance of 23 nt csR-trnA-2 csRNAs remarkably increased (Figure 7E), consistent with the deep sequencing result. Like rRNA-derived csRNAs, tRNA-derived csRNAs are affected by heat stress.

**Table 2:** Heat response of tRNA-derived csRNAs

*Group*	*tRNA*	*Heat response*	***Percentage (%) ***^***a***^	
			*MT*	*HT*
I	Ala,Asp,Phe,Lys,Leu,Gln,Thr,Val	Shorter csRNAs increased, and longer csRNAs decreased ^b^	81	73

II	Cys,Glu,Ile,Met,Asn,Pro,Ser,Trp	Shorter csRNAs increased, and longer csRNAs unchanged	12	20

III	His,Arg	Longer csRNAs decreased, and shorter csRNAs unchanged	5	5

IV	Gly	18 nt csRNAs decreased, and the others unchanged	2	2

^a^: Percentage for each group in total abundance of tRNA-derived csRNAs.

^b^: The shorter csRNAs ranged from 15 to 25 nt in length, and the longer csRNAs were from 29 to 32 nt.

In addition to the above-mentioned origins, small RNAs of 21~24 nt from mRNAs and igRNAs increased in HT seedlings compared to those in the control lines (Additional File 4E-H), and those showing relatively high abundances were verified by a microRNA microarray analysis (Additional File 9).

### Validation of csRNAs in chloroplasts

**Figure 8:**
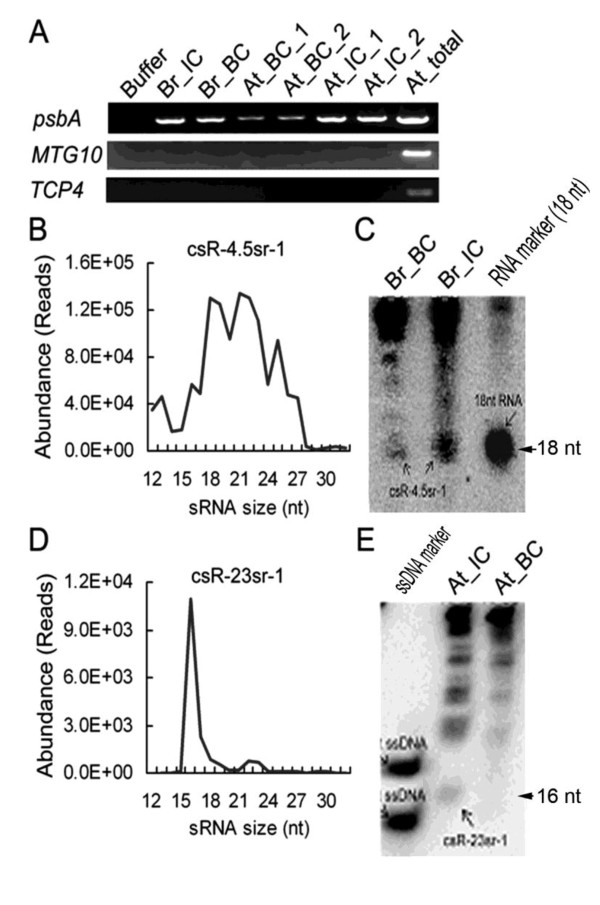
**Validation of csRNAs in chloroplasts**. (**A**) RT-PCR analysis of *ATCG00020 *(chloroplast gene encoding chloroplast psbA protein), *At4g10760 *(nuclear gene encoding chloroplast MTA protein) and *At4g15030 *(nuclear gene encoding the nuclear TCP4 protein). (**B**) and (**C**) Size distribution and Northern blotting of csR-4.5sr-1 in Chinese cabbage. (**D**) and (**E**) Size distribution and Northern blotting of csR-23sr-1 in *Arabidopsis*. Buffer: chloroplast isolation buffer mentioned in [47]; Br_IC: intact chloroplasts of Chinese cabbage. Br_BC: broken chloroplasts of Chinese cabbage; At_IC: intact chloroplasts of Arabidopsis; At_BC: broken chloroplasts of Arabidopsis; At_total: total RNA samples extracted from Arabidopsis.

To investigate whether the csRNAs are located in the chloroplast, we isolated the highly purified chloroplasts of Chinese cabbage and *Arabidopsis *and performed Northern blotting. For comparison, the RNA samples from the intact and broken chloroplasts were separated. The lack of nuclear RNA contamination was confirmed by PCR after reverse transcription, using primers designed for (i) *At4g10760 *(the nuclear gene encoding the chloroplast MTA protein) and (ii) *At4g15030 *(encoding the nuclear TCP4 protein) gene sequences (Figure 8A). csRNA-4.5sr-1 (sequence in Additional File 9) was the most abundant csRNA family, and its csRNAs were derived from the 3' end of the 4.5S rRNA in chloroplasts with its 3' termini located -1 to +1 nt relative to the last nucleotide of the rRNA. In the csRNA datasets of Chinese cabbage, three abundance peaks at ~18, 22 and 25 nt were displayed consistently for the HT and MT seedlings (Figure 8B). Using Northern blotting analyses, the 18 nt csRNAs and the other csRNAs belonging to csRNA-4.5sr-1 were identified in the intact and broken chloroplast RNAs. Nevertheless, the csRNA-4.5sr-1 csRNAs in the broken chloroplast were less abundant than in the intact chloroplasts (Figure 8C). Similarly, the csR-23sr-1 csRNAs in the broken chloroplasts of *Arabidopsis *were less abundant than in the intact chloroplasts (Figure 8E). In addition, amount of ~16 nt csR-23sr-1 csRNAs is in agreement with our *Arabidopsis *deep sequencing results (Figure 8D). We noticed several RNA molecules of 80-200 nt in size that might be the multiple precursors (Figure 8C and 8E).

Apart from the observations above, one small RNA core sequence, csRNA-ig-1, was represented by a large number of reads at an average size of 22 nt in each of the Chinese cabbage csRNA datasets and a specific 24 nt size in the *Arabidopsis *chloroplast dataset, which mapped to one intergenic RNA locus but nowhere else. This small RNA sequence is identical to the Ntc-2 small non-coding RNA reported and verified by Lung and colleagues in their tobacco chloroplast study [10]. Interestingly, csRNA-ig-1 was induced under heat stress (Additional Files 8 and 9).

### Putative structure of csRNAs and their precursors

**Figure 9:**
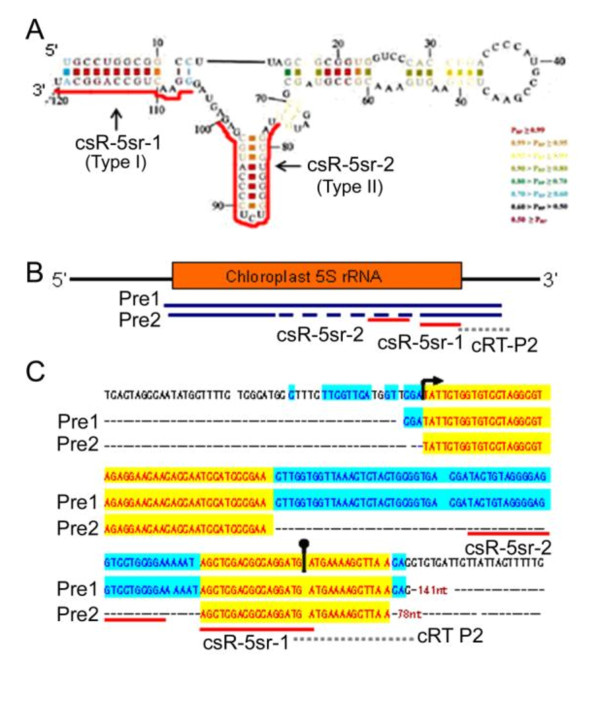
**The predicted secondary structures and putative precursors of csR-5sr-1 and csR-5sr-2**. (**A**) The prediction of csR-5sr-1 and csR-5sr-2 on the secondary structure of 5S rRNA from *E. coli*, according to their location numbers on Chinese cabbage chloroplast 5S rRNA. (**B**) The sequenced transcripts of putative precursor of csR-5sr-1. The dashed line indicates the internal deletion. (**C**) Alignment of the putative precursors of csR-5sr-1 with 5S rDNA. The arrow and round-head arrow directed the first and last nucleotides of 5S rRNA, respectively. csR-5sr-1 sequence is underlined in red color. The dotted lines indicate the position of PCR primer 2.

To associate small RNAs with the folded conformations of RNA, we analyzed the secondary structure of some rRNA and tRNA molecules. According to the secondary structures of 5S and 4.5S rRNA from *E. coli *[31,32] and 16S rRNA from *Haloferax volcanii *[33], we simulated the secondary structure of some rRNAs-csRNAs of Chinese cabbage with high abundance. csR-5sr-1 and csR-5sr-2, two of the major csRNA sets derived from the Chinese cabbage chloroplast 5S rRNA (Additional File 9), matched a "single-stem-with-bulge" frame (type I) and a "self-hairpin" frame (type II) of 5S rRNA (Figure 9A). The parallel analysis of the csRNAs from 4.5S RNA of *E. coli *indicated that two csRNAs derived from 4.5S rRNA and three csRNAs derived from 16S RNA were located in "single-stem-with-bulge" frame (Additional File 11A). Meanwhile, three of the other csRNAs from 16S rRNA present a potential secondary structure of "self-hairpin" (Additional File 9). csR-16sr-1 is the most abundant csRNA from 16S rRNA. This csRNA and csR-16sr-2 exhibited "two-stem-with-hinge" frame (Additional File 11B).

tRNA-derived small RNAs were mostly identified by starting from the first nucleotide of the mature tRNA sequences. Compared to the cloverleaf structures of the tRNA, this position-specificity resulted in a 25 nt csRNA that had the exact same sequence as the acceptor arm from the 5' end, including the D-arm and D-loop of the tRNA; a few of nucleotides of csRNAs of 27~31 nt were matched to the stem of anticodon arm; and even a 36 nt csRNA was paired with the complete anticodon (Additional File 11C). Zhang et al. [27] reported a class of ~58-60 nt non-coding RNAs in the phloem sap of pumpkin, whose cleavage sites fell in the anticodon and D arm of tRNA. csRNAs of Chinese cabbage are much shorter than these non-coding RNAs, but both are generated in anticodon or D loop of tRNA.

To investigate the conceivable precursors of the csRNA sequences, circular RT-PCR was carried out. Two different RNA fragments of csR-5sr-1 precursor were isolated and sequenced (Figure 9B). Pre-1 was 141 nt in length, 20 nt longer than mature sequence of 5S rRNA, with a 3 nt extension to the 5' ends, and a 15 nt extension to 3' ends (Figure 9C). Pre-2 was 77 nt in length, with a 13 nt extension outer of 3' ends, but with a 58 nt internal deletion. According to the secondary structure deduced from *E. coli *(Figure 9A), Pre-1 and Pre-2 are the putative precursors of csR-5sr-1.

### Relationship between csRNAs and the putative targets

**Figure 10:**
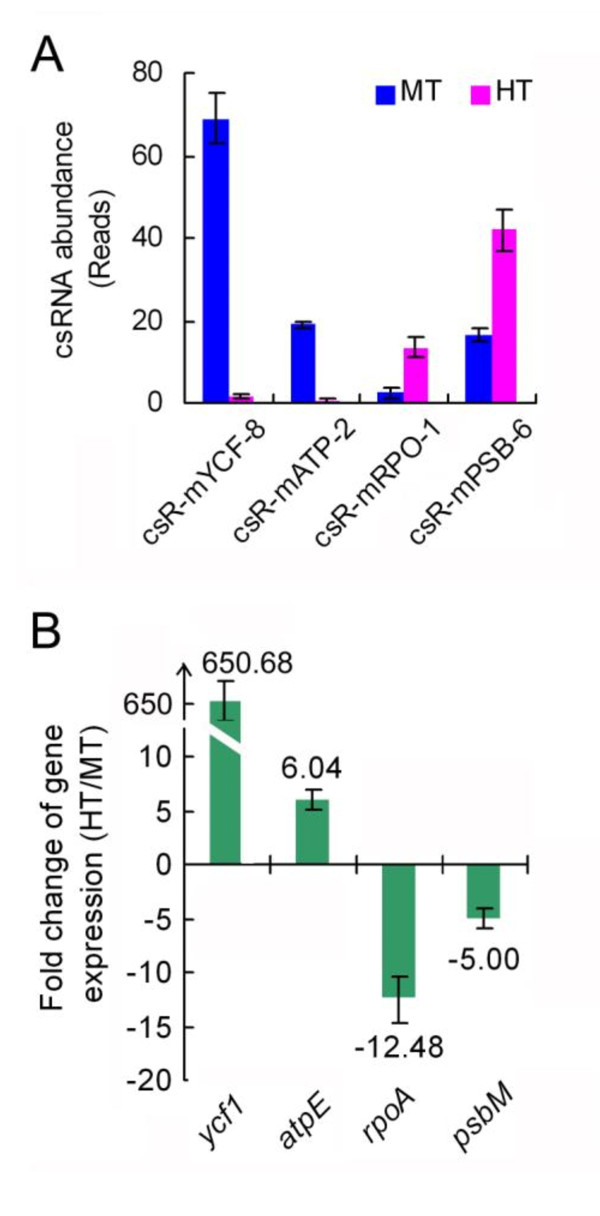
**Expression analysis of the four putative target genes of csRNAs**. (**A**) A csRNA abundance comparison between HT and MT samples from Chinese cabbage deep-sequencing datasets. (**B**) Fold changes of putative target gene expression.

To detect the functions of csRNAs, we searched for the transcripts of the coding genes that are complemented by the csRNAs responsive to heat stress. csR-mYCF-8, a csRNA family of Chinese cabbage (Additional File 9) that declined under heat treatment (Figure 10A), perfectly matched to the chloroplast *ycf1 *gene. Using adjacent sequences as primers, the real-time PCR showed that expression of gene dramatically increased in the HT seedlings compared with those in the MT seedlings (Figure 10B), implying a silencing role of csR-mYCF-8 csRNAs in expression of *ycf1 *gene. Similarly, *atpE *was a putative target of csR-mATP-2, which was up-regulated in the HT seedlings, just opposite to the reduced abundance of the csR-mATP-2 csRNAs (Figure 10). Other than *ycf1 *and *atpE, rpoA *and *psbM *were other two possible targets paired with csR-RPO-1 and csR-mPSB-6 csRNAs, respectively (Additional File 9). As shown by small RNA deep-sequencing and microRNA microarray, these two csRNAs were remarkably increased by heat stress in the HT seedlings (Figure 10A). Opposite to this, the expression of the *rpoA *and *psbM *gene was down-regulated in the HT seedlings (Figure 10B). These consistent results revealed that csRNAs play certain roles in regulation of gene expression.

## Discussion

### Chloroplast RNA generates a novel subset of small RNAs

Although studies in recent years have reported the existence of small non-coding RNAs in DNA-containing cellular organelles [10-12], a genome-wide chloroplast profiling study is not reported yet, and therefore, the expression patterns, biological functions and biogenesis pathways of stable small RNA species remain largely unknown. From small RNA libraries of both Chinese cabbage and *Arabidopsis*, we selected two classes of the chloroplast-related small RNAs (csRNAs). By comparing chloroplast-specific small RNAs with csRNAs in *Arabidopsis*, we showed that most of the small RNAs in the csRNA dataset were derived exclusively from the chloroplast genome rather than the nuclear or mitochondrial genomes; thus, csRNA dataset may represent the features of chloroplast-derived small RNAs. Using highly purified chloroplast RNA samples, we confirmed by Northern blotting that most of the csRNAs were produced and accumulated inside chloroplasts.

Among csRNAs from the ~150 kb sequence of the chloroplast genome, about 90% were mapped to chloroplast rRNA and tRNA loci. Although fragments from rRNA and tRNA are regularly considered to be degradation contaminants, we present several clues suggesting a different viewpoint: a) Most of the tRNA/rRNA-derived csRNAs were associated with the 5' ends of tRNAs or 3' ends of rRNAs, respectively, and were even precisely aligned to the first nucleotides of mature tRNA, representing specific positions in the chloroplast genome. b) Despite wide varieties in size, the csRNA families exhibited distinct size distributions. A csRNA family is usually predominated by small RNAs of one or two determinate sizes, although it is classified as a group of small RNAs, constant in their core sequence and varying in the 5' or 3' extender length. c) Some extra nucleotides ahead and behind the matured RNA sequences were revealed from the putative csRNA precursors (such as Pre1 and Pre2 of csR-5sr-1). Considering the high abundance and end-specificity of some csRNA families such as csR-5sr-1, the csRNAs may not be the degradation products. d) csRNAs not only originated from the region corresponding to the mature RNAs but also from the externally and internally transcribed spacer regions, indicating that their biogenesis may require un-transcribed precursors or intermediate products. Collectively, our data suggest an undiscovered biogenesis pathway of small RNAs from RNA processing that differs from the existing knowledge about the single nucleotide decay from the end of a DNA or RNA chain by exonucleases.

### csRNAs are highly sensitive to high temperature

Comparing the csRNAs from heat-treated samples with those from the non-stressed control group, the most remarkable change was that the rRNA-derived csRNAs declined significantly in the HT dataset to only ~50% of their original abundance shown in the MT dataset. However, the reduction in the 5S rRNA-derived csRNAs may due to the decease of chloroplast rRNA/rDNA by heat stress, as reported in maize chloroplast rRNA [34] and black mustard (*Brassica nigra*) [35]. The rRNA transcripts could be the sources of not only mature rRNA, but also csRNA, as most chloroplast small RNA sequences were identical to the sense strand of chloroplast DNA. Under these circumstances, csRNA biogenesis from rRNA may be passively controlled by the quantity of total transcripts. However, other possibilities still exist: the csRNAs may decrease to a certain extent in an initiative manner compared to the changes in transcripts. It is challenging to compare the changes in transcripts, mature rRNAs and csRNAs directly. Since the relations between two of the three are still indeterminable, it is unknown whether there is another factor involved in their processing pathways. One possible alternative model for the regulation of RNA levels by csRNA is as follows: polycistronic precursors produce both csRNAs and mature RNAs; as a result, fewer csRNAs lead to more mature rRNAs processed from the precursor, and less interference caused by the base-pairing competition between fragments with the same nucleotide sequences. In addition, a previous study involving 5S rDNA-derived siRNAs in DDM1 and MET1-deficient mutants suggested a role of siRNAs in 5S rDNA chromatin organisation [36], which may hint to a similar role for the 5S rRNA-originated csRNAs in the chloroplast.

Among the tRNA-derived csRNAs, the number of shorter csRNAs (15-25nt) was reduced, while the number of longer csRNAs (29-32nt) was increased. Interestingly, the 3' termini of the shorter and longer csRNAs mapped within the D arm and anticodon arm, respectively, indicating that the tRNA cleavage sites were located in the middle. tRNA halves, corresponding to the 5' or 3' sequences of the tRNAs, derived from mature tRNAs cleaved at their anticodon loops by an unidentified RNase, were able to inhibit translation and potentially transmit long-distance signals in plant phloem sap [27]. Processed tRNA halves were also detected in prokaryotes, fungi, yeast, *Arabidopsis*, and human cells, under various stresses, such as early amino acid starvation or oxidative stress [37-40]. Although we do not yet know whether those csRNAs associated with tRNA 5' ends in our datasets are functional 5' halves or not, there is a possibility that they are generated by a similar pathway in the chloroplast, reflecting an ancient mechanism centrally associated with stress conditions.

In contrast to small RNAs from rRNA, tRNA and mRNA, the igRNA-derived csRNAs increased by 30% after heat treatment.

### csRNA may play roles in plant heat resistance

Leaf etiolation is one of the major indicators of plant sensitivity or tolerance to high temperature. When the intracellular environment is exposed to moderate heat stress, the structure of the thylakoid membrane is changed and cyclic electron flow around photo system I is increased, resulting in massive photo-oxidative stress and the concomitant release of highly cyto-toxic free radicals and reactive oxygen species [41,42]. When the subcellular structures of the chloroplast are damaged by heat stress, photosynthetic capacity is greatly reduced [41]. This change is usually concomitant with leaf etiolation. For the heat-resistant genotypes of Chinese cabbage, leaf etiolation appears later or only slightly compared with the heat-sensitive genotypes (data not shown). In the HT seedlings, most of the csRNAs showed reduced abundances 1 hour after the 46°C heat treatment, the time period before the appearance of leaf etiolation. One hypothesis is that the heat-responsive csRNAs play roles in the maintenance of subcellular structures and photosynthetic capacity of the chloroplast. The csRNAs derived from tRNAs are possibly play therole similar to the reported tRNA halves under various stress conditions in prokaryotes, fungi, yeast, *Arabidopsis *and human cells [27,38-40]. When seedlings are exposed to high temperature, the biogenesis of the heat-responsive csRNA is affected. The change in abundance of some csRNAs affects the putative target genes, which may play an important role in plant resistance to heat stress.

Plant chloroplasts are closely related to leaf development, while the latter is regulated by many miRNAs and small RNAs [43-46]. However, it remains to be elucidated how the novel csRNAs are cooperating with the canonical miRNAs in leaf development after exposure to high temperatures and if they are affecting each other. To address the roles of these heat-responsive csRNAs in plant heat resistance, a study has been planned to compare the gene expression patterns of heat-resistant cultivars of Chinese cabbage with the heat-sensitive genotypes. This study could provide insight into understanding the molecular mechanism behind csRNA-mediated heat resistance and a fast and efficient way to improve the heat resistance of Chinese cabbage and other important crops.

## Conclusion

The chloroplast is an important organelle that contains a plenty of small RNAs. Our results show that many members of csRNA families were highly sensitive to heat stress. Some csRNAs respond to heat stress by silencing target genes. We suggest that proper temperature is important for production of chloroplast small RNA, which are associated with plant resistance to heat stress.

## Methods

### Plant materials and growth conditions

Wu-11, a heat-sensitive inbred line of non-heading Chinese cabbage (*B. rapa ssp. chinensis*), was used in this study, unless indicated otherwise. The seeds were sown in pots and germinated in an incubator. After one week, the seedlings were transplanted to the growth chambers in Phytotron of SIPPE (Shanghai Institute of Plant Physiology and Ecology) and grown at 22°C with 16 h of light per day. Three-week-old seedlings were autoclaved at 46°C (HT, high temperature) and 22°C (MT, moderate temperature) in two water bath kettles, respectively, for one hour. After heat treatment, some of the seedlings were used for RNA isolation while the rest were transferred to growth chambers for further growth.

The seeds of *Arabidopsis thaliana *were surface-sterilized in 70% ethanol for 1 min, followed by 0.1% HgCl_2 _for 10 min, washed four times in sterile distilled water and plated in molten 0.1% water agar on top of solid 1% sugar MS_0 _medium. Plates were sealed with Parafilm, incubated at 4°C in darkness for 2-3 days and then moved to a growth chamber at 22°C with 16 h of light per day.

### RNA extraction and quantity detection

After heat treatment, the above-ground portions of the seedlings were harvested, quick-frozen immediately with liquid nitrogen and stored at -80°C. Each treatment comprised 10 seedling samples that were divided into two replicates. Total RNAs were extracted using TRIzol Reagent (Invitrogen). RNA concentration was measured by Eppendorf Biophotometer 6121. DNA was removed by digestion using TURBO DNA-free kit (Ambion).

### Chloroplast isolation

Chloroplasts were isolated from seedlings using a Percoll (GE, 17089101) gradient-based method [47]. The intactness of chloroplast was estimated by microscopic examination (Olympus BX51 wide-field microscope with differential interference contrast) and Hill reaction [48].

### Small RNA deep sequencing and sequence analysis

Chinese cabbage and *Arabidopsis *total RNA samples were sent to Keygene N.V., Wageningen, the Netherlands and Zhejiang-California Nanosystems Institute, China respectively, where the samples were prepared and sequenced on Illumina GAII sequencer, according to the manufacturer's protocol. The adaptor sequences in Illumina sequencing reads were removed using "vectorship" in the EMBOSS package. The small RNA with length of 9~36 nt were mapped to chloroplast genomes (GenBank accession number: DQ231548) and nuclear genomic sequences (BRAD, Brassica Database, http://brassicadb.org/brad/) of Chinese cabbage (*Brassica rapa *ssp. *chinensis*) and nuclear, chloroplast, mitochondrial genomes of *Arabidopsis *(http://www.arabidopsis.org). All data from different samples were normalized before comparison analysis.

### Northern blotting analysis

RNA (20 μg) was fractionated on a 19% denaturing poly-acrylamide gel (PAGE), transferred to a Hybond-XL membrane (Amersham biosciences-GE healthcare) by capillary transfer using 20 × SSC buffer, and fixed by UV-crosslinking. Pre-hybridization was carried out at 42°C for 2 × 30 min using PrefectHyb Hybridization Buffer (Sigma). Oligo-nucleotide DNA probes, were labeled with γ^32^P ATP (5000Ci/mmol) using a T4 polynucleotide kinase (Roche). Hybridization was performed overnight at 42°C. Sizes of csRNA molecules were estimated using oligo-nucleotide DNA/RNA sense-probes with sequences same with csRNAs. In addition to equivalent RNA loading (20 μg detected by Eppendorf Biophotometer 6121), U6 spliceosomal RNA was used as an RNA loading control for total RNA sample.

### Quantitative real-time reverse transcription PCR

10 mg of each Chinese cabbage total RNA sample were treated twice with 4 U of TURBO DNase (Ambion) for 30 min at 37°C. For cDNA synthesis, 200 ng RNA were reverse transcribed using SuperScript™ First-Strand Synthesis System with random hexamers (Invitrogen) according to manufacturer's recommendations. A MyiQ2 real-time PCR detection system (Bio-rad) was used for qPCR (2 min at 50°C, 10 min at 95°C, 40 × (15 s at 95 °C, 1 min at 58°C)) with the Power SYBR Green kit (Applied Biosystems; 10 μl master mix, 5 μl cDNA (diluted 1: 10 for chloroplast *atpE, psbM, rpoA *and *ycf1*; and 1: 5 for Chinese cabbage *UBQ5*), 1 μl of forward and reverse primer (1 μM each), 3 μl H_2_O; *atpE *primers: S-tttgctgagcttcttgtgga, A- cgaatcgaattgtttgggat; *psbM *primer: S-ggaacgagaatgaagagtgc, A-tcccgagatattccaaagaa; *rpoA *primer: S-agattctgggaggcaattct, A-aagcacttcatcaagcctcc; *ycf1 *primers: S-accaatccaacccatttcat, A-ccaagttcaatgttagccaga; *UBQ5 *primers: S-tccgtccaccttgtagaactg, A-tgaaaaccctaacggggaaa). At least four biological replications were detected for each gene. All reactions were done in triplicate, and for each reaction a 'no RT' control was included. Data analysis was performed with the iCycler IQ real-time detection system (Bio-Rad), with automatic Ct and baseline setting and *UBQ5 *mRNA concentrations as internal control. Relative transcript quantities were calculated with the ΔΔCt method.

### Circular reverse transcription PCR (cRT-PCR)

The precise 5' and 3' ends of the csRNA precursors were determined by circular RT-PCR. The DNase treated chloroplast RNAs were incubated with 40U of T4 RNA ligase (New England Biolabs) according to manufacturer's protocol, which could generate circular RNAs with the 5' and 3' ends joined together. After phenol-chloroform extraction, single-strand cDNAs were synthesized using Revertra Ace (TRT-101, Toyobo) and specific reverse primer. The sequences of the reverse transcript products were complementary to the small RNA precursors, with breakpoints at the ends. PCR amplification were then carried out using a forward primer (cRT Primer 1: gctcgacgccaggatg) as same sequence as csR-5sr-1, and a reverse primer (cRT Primer 2: gtgttaagcttttcat) which complement to the DNA sequence following the binding site of csRNA (indicated in Figure 9). PCR amplification consisted of 30 cycles of 30s duration at 94°C, 30s at 48°C and 30s at 72°C. All cRT-PCR products were purified by MagExtractor (NPK-600, Toyobo), and then ligased into pMD18-T vector (D101A, Takara) for sequencing.

## Abbreviations

cRT-PCR: circular semi-quantitative reverse transcription polymerase chain reaction; csRNA: chloroplast small RNA; DDM1: decrease in DNA methylation 1; dsRNA: double-stranded RNAs; HT: high temperature; igRNA: intergenic RNA; MET1: cytosine methyltransferase mutant 1; mRNA: messenger RNA; MT: moderate temperature; ncRNA: non-coding RNA; nt: nucleotide; rRNA: ribosomal RNA; siRNA: small interfering RNA; snoRNA: small nucleolar RNAs; tRNA: transfer RNA.

## Authors' contributions

The work presented here was carried out in collaboration between all authors. HYK defined the research theme and directed the experiments. WL carried out the chloroplast isolating, small RNAs, precursors and target genes detecting, some of the bioinformatics analyses, and participated in manuscript writing. YX and LYZ constructed the small RNA libraries and performed most of the bioinformatics analyses. WH, PM and DRM co-worked on the small RNA sequencing. HYK, PM, YX and WH also revised this paper. All authors have contributed to, seen and approved the manuscript.

## Supplementary Material

Additional file 1 **Abundance of sequenced small RNAs matched to genomes of *Arabidopsis *thaliana (*Landsberg *erecta ecotype)**. Click here for file

Additional file 2 **Comparison analyses of *Arabidopsis *chloroplast-related small RNAs (Related) and chloroplast-specific small RNAs (Specific)**. Click here for file

Additional file 3 **RNA origin analysis of Chinese cabbage csRNAs**. Click here for file

Additional file 4 **Size distribution of csRNAs in the HT seedlings**. Click here for file

Additional file 5 **csRNAs of csR-5sr-1 family**. Click here for file

Additional file 6 **The position analysis of the 3' ends of rRNA-derived csRNAs of Chinese cabbage**. Click here for file

Additional file 7 **The csRNAs that were reduced in the HT lines (less than 20%)**. Click here for file

Additional file 8 **The csRNAs that were increased in the HT lines with 5 folds**. Click here for file

Additional file 9 **csRNAs detected by microRNA microarray analysis**. Click here for file

Additional file 10 **rRNA-derived csRNAs were drastically reduced in the HT seedlings**. Click here for file

Additional file 11 **The predicted structures of some abundant csRNAs from 4.5S and 16S rRNAs, and from tRNA-Asp**. Click here for file
