# Supplementary material for: A novel class of heat-responsive small RNAs derived from the chloroplast genome of Chinese cabbage (Brassica rapa)
Source: BMC Genomics. 2011 Jun 3;12:289. doi: 10.1186/1471-2164-12-289 (PMC3126784; doi:10.1186/1471-2164-12-289)
Supplement: Additional file 1 — Abundance of sequenced small RNAs matched to genomes of Arabidopsis thaliana (Landsberg erecta ecotype). [file 1471-2164-12-289-S1.DOC]

Additional File 1. Abundance of sequenced small RNAs matched to genomes of *Arabidopsis* thaliana (Landsberg erecta ecotype).

| **Genome sources of small RNAs** | **Number of unique reads** | **Abundance of total reads** |
| --- | --- | --- |
| Nuclear genome only | 1444271 (88.8%) | 7184913 (89.7%) |
| Chloroplast genome only ^a^ | 126545 (7.8%) | 667,355 (8.4%) |
| Mitochondria genome only | 14117 (0.9%) | 23199 (0.3%) |
| Common in nuclear and chloroplast genomes ^b^ | 15432 (0.9%) | 44109 (0.5%) |
| Common in nuclear and mitochondria genomes | 10383 (0.6%) | 16906 (0.2%) |
| Common in chloroplast and mitochondria genomes ^c^ | 9543 (0.6%) | 60052 (0.7%) |
| Common in nuclear, chloroplast & mitochondria genomes ^d^ | 6230 (0.4%) | 15756 (0.2%) |
| **Total** | **1626521** | **8012290** |

Note, ^a^ = *Arabidopsis* chloroplast-specific small RNAs, and ^a^+^b^+^c^+^d^ = *Arabidopsis* chloroplast-related small RNAs.
