# Supplementary material for: A novel class of heat-responsive small RNAs derived from the chloroplast genome of Chinese cabbage (Brassica rapa)
Source: BMC Genomics. 2011 Jun 3;12:289. doi: 10.1186/1471-2164-12-289 (PMC3126784; doi:10.1186/1471-2164-12-289)
Supplement: Additional file 2 — Comparison analyses of Arabidopsis chloroplast-related small RNAs (Related) and chloroplast-specific small RNAs (Specific). [file 1471-2164-12-289-S2.DOC]

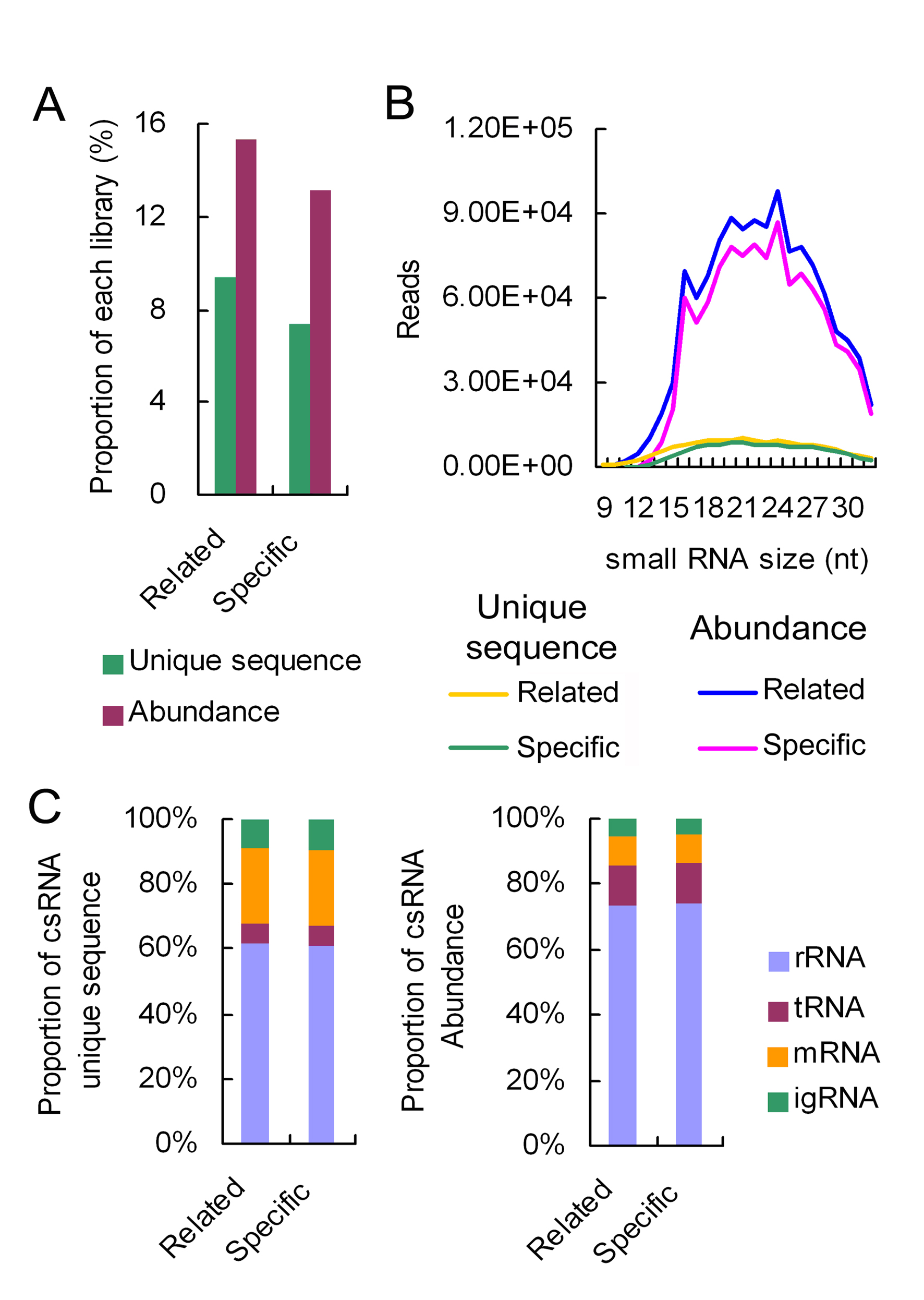


Additional File 2. Comparison of *Arabidopsis* chloroplast-related small RNAs (Related) and chloroplast-specific small RNAs (Specific).

(**A**) Proportion of chloroplast small RNA population in the total dataset.

(**B**) Size distribution.

(**C**) Chloroplast RNA origin.
