# Supplementary material for: A novel class of heat-responsive small RNAs derived from the chloroplast genome of Chinese cabbage (Brassica rapa)
Source: BMC Genomics. 2011 Jun 3;12:289. doi: 10.1186/1471-2164-12-289 (PMC3126784; doi:10.1186/1471-2164-12-289)
Supplement: Additional file 3 — RNA origin analysis of Chinese cabbage csRNAs. [file 1471-2164-12-289-S3.DOC]

Additional File 3. RNA origin analysis of Chinese cabbage csRNAs.

| ***Dataset*** | ***Origin*** | ***Unique sequence*** * | ***Abundance*** |
| --- | --- | --- | --- |
| MT | rRNA | 44,285 | 1,266,980 |
|  | tRNA | 12,466 | 712,273 |
|  | mRNA | 23,277 | 49,018 |
|  | igRNA | 10,169 | 68,238 |
|  | **Sum** | **90,197 ^a^** | **2,096,509 ^b^** |
| HT | rRNA | 27,338 | 557,768 |
|  | tRNA | 10,582 | 560,170 |
|  | mRNA | 14,523 | 32,450 |
|  | igRNA | 6,909 | 75,422 |
|  | **Sum** | **59,352 ^c^** | **1,225,810 ^d^** |

*: The numbers represent the quantities of sequenced reads before normalization.

The duplicated calculation of 319 (**^a^**), 91 (**^c^**), 1431 (**^b^**) and 272 (**^d^**) unique sequences was included.
