# Supplementary figures and images for: A novel class of heat-responsive small RNAs derived from the chloroplast genome of Chinese cabbage (Brassica rapa)
Source: BMC Genomics. 2011 Jun 3;12:289. doi: 10.1186/1471-2164-12-289 (PMC3126784; doi:10.1186/1471-2164-12-289)

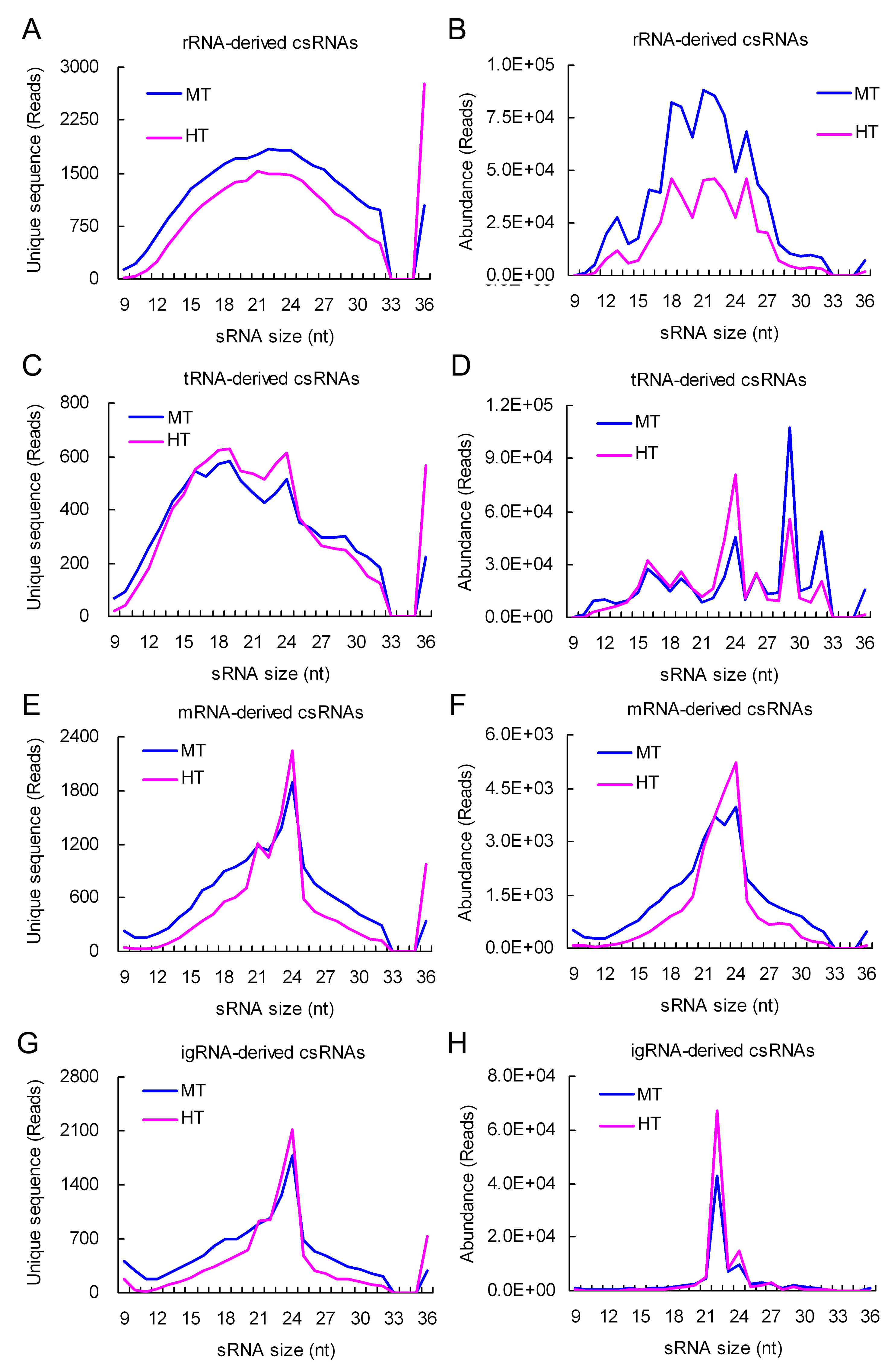


Additional File 4. Size distribution of csRNAs in the HT seedlings.

Supplement: Additional file 4 — Size distribution of csRNAs in the HT seedlings. [file 1471-2164-12-289-S4.DOC]
