# Supplementary material for: A novel class of heat-responsive small RNAs derived from the chloroplast genome of Chinese cabbage (Brassica rapa)
Source: BMC Genomics. 2011 Jun 3;12:289. doi: 10.1186/1471-2164-12-289 (PMC3126784; doi:10.1186/1471-2164-12-289)
Supplement: Additional file 6 — The position analysis of the 3' ends of rRNA-derived csRNAs of Chinese cabbage. [file 1471-2164-12-289-S6.DOC]

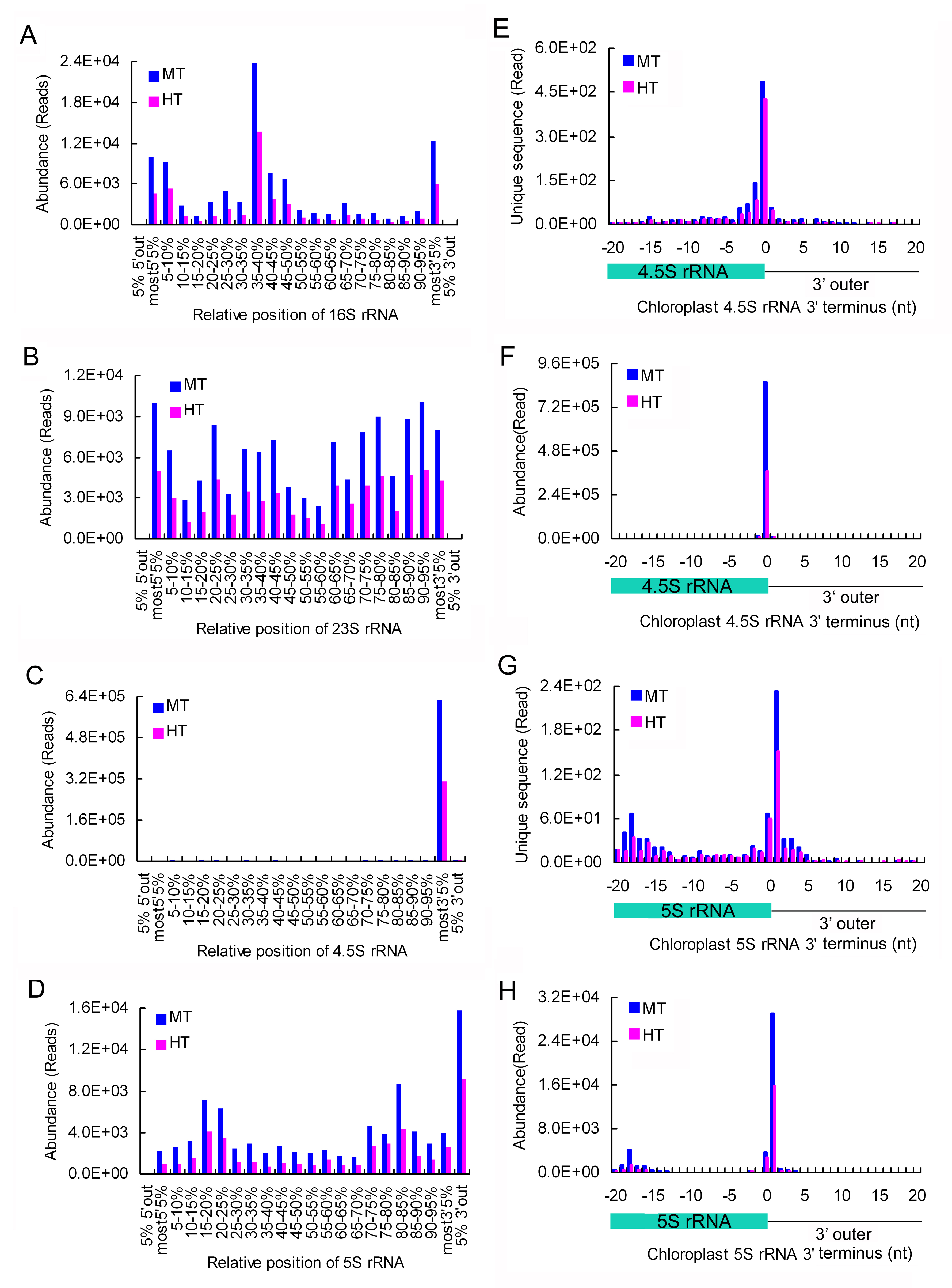


Additional File 6. The position analysis of the 3’ ends of rRNA-derived csRNAs of Chinese cabbage.

(**A**), (**B**), (**C**) and (**D**) Position-of-origin analysis of the 3’ ends of csRNAs from 16S rRNA, 23S rRNA, 4.5S rRNA and 5S rRNA. rRNA sequences were divided into 5% blocks from the 5’ to the 3’ ends, and the 3’ ends of csRNAs were plotted into them. The flanking sequences outer of each ends were calculated proportionally.

(**E**) and (**F**) Unique sequence and abundance at the 3’ ends of csRNAs relative to the last nucleotide of mature 4.5S rRNA. Position 0 corresponds to the last transcribed nucleotide of mature 4.5S rRNA sequence.

(**G**) and (**H**) Unique sequence and abundance at the 3’ ends’ of csRNAs relative to the last nucleotide of mature 5S rRNA. Position 0 corresponds to the last transcribed nucleotide of mature 5S rRNA sequence.
