# Supplementary material for: A novel class of heat-responsive small RNAs derived from the chloroplast genome of Chinese cabbage (Brassica rapa)
Source: BMC Genomics. 2011 Jun 3;12:289. doi: 10.1186/1471-2164-12-289 (PMC3126784; doi:10.1186/1471-2164-12-289)
Supplement: Additional file 10 — rRNA-derived csRNAs were drastically reduced in the HT seedlings. [file 1471-2164-12-289-S10.DOC]

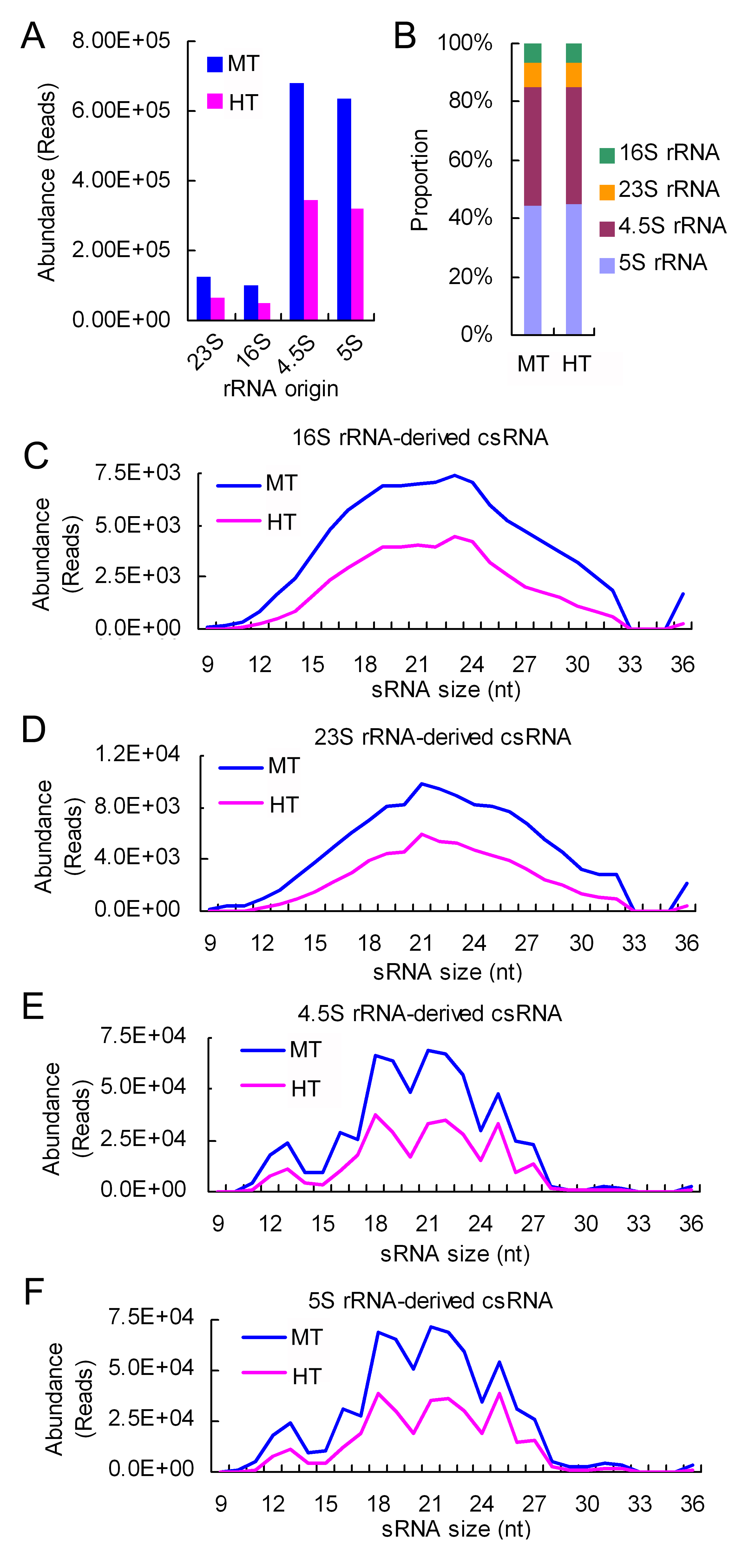


Additional File 10. rRNA-derived csRNAs were drastically reduced in HT seedlings.

(**A**) Abundance of csRNA.

(**B**) Proportion of csRNAs.

(**C**-**F**) Size distribution of csRNAs from Chinese cabbage chloroplast 16S (**C**), 23S (**D**), 4.5S (**E**) and 5S (**F**) rRNA.
