# Supplementary material for: A novel class of heat-responsive small RNAs derived from the chloroplast genome of Chinese cabbage (Brassica rapa)
Source: BMC Genomics. 2011 Jun 3;12:289. doi: 10.1186/1471-2164-12-289 (PMC3126784; doi:10.1186/1471-2164-12-289)
Supplement: Additional file 11 — The predicted structures of some abundant csRNAs from 4.5S and 16S rRNAs, and from tRNA-Asp. [file 1471-2164-12-289-S11.DOC]

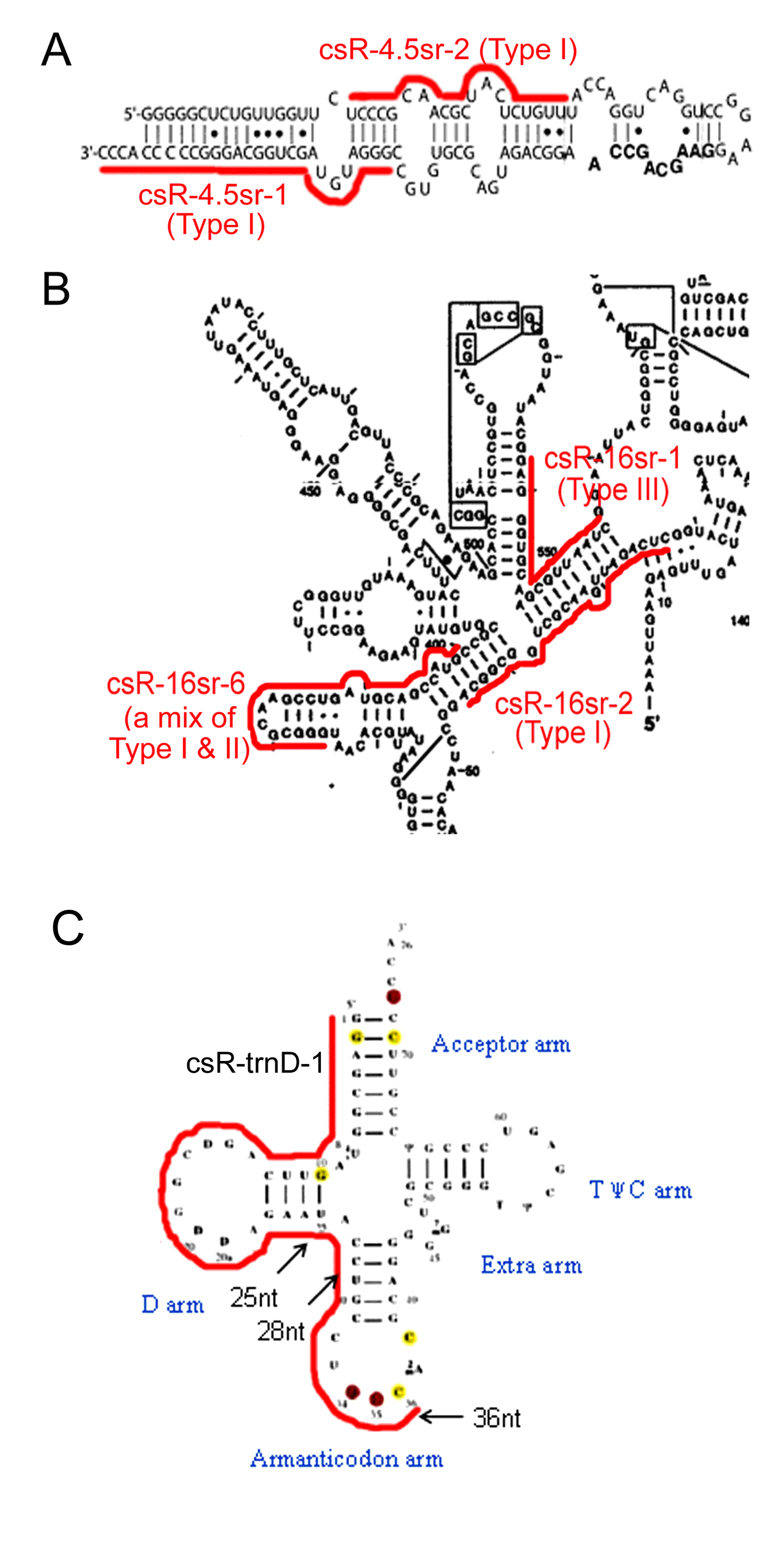


Additional File 11. The predicted structures of some abundant csRNAs from Chinese cabbage chloroplast 4.5S and 16S rRNAs, and from tRNA-D.

(**A**) The predicted secondary structures of csR-4.5sr-1 and csR-4.5sr-2 according to *E. coli*.

(**B**) The predicted secondary structures of csR-16sr-1, csR-16sr-2 and csR-16sr-6 according to *Haloferax volcanii* 16S rRNA.

(**C**) The predicted structure of csRNAs on tRNA-D secondary structures.
